# Fungal detection, diagnostic approaches, and antifungal susceptibility in diabetic foot ulcers: a systematic review of recent evidence

**DOI:** 10.3389/ffunb.2026.1915109

**Published:** 2026-09-04

**Authors:** Salma M. Alsayed, Aiah M. Khateb, Muhammad Yasir, Bayan Bogary, Ahmed M. Aljabri, Ahmad Al Atrouni, Rnda Baabdullah, Ahmed Afandi, Safaa A. Turkistani, Wesam A. Abduljabbar, Mohammed Salleh M. Ardawi, Esam I. Azhar

**Affiliations:** 1 Department of Biological Sciences, King Abdulaziz University Faculty of Sciences, Jeddah, Saudi Arabia; 2 Special Infectious Agents Unit, King Fahd Center for Medical Research, King Abdulaziz University, Jeddah, Saudi Arabia; 3 Department of Clinical Laboratory Sciences, Faculty of Medicine, College of Applied Medical Sciences, Taibah University, Madinah, Saudi Arabia; 4 Pathological Sciences Department- MBBS Program, Fakeeh Care Group, Fakeeh College for Medical Sciences, Jeddah, Saudi Arabia; 5 Department of Surgery, King Fahad Armed Forces Hospital, Jeddah, Saudi Arabia; 6 Microbiology Laboratory, Dr. Soliman Fakeeh Hospital, Fakeeh Care Group, Jeddah, Saudi Arabia; 7 Medical Laboratory Sciences Department, Medical Laboratory Sciences (MLS) Program, Fakeeh Care Group, Fakeeh College for Medical Sciences, Jeddah, Saudi Arabia; 8 Medical Laboratory Sciences Department, Faculty of Applied Medical Sciences, King Abdulaziz University, Jeddah, Saudi Arabia

**Keywords:** antifungal, *Aspergillus*, *Candida*, diabetic foot ulcer, diagnosis, fungal infection, fungi, polymicrobial infection

## Abstract

**Background:**

Fungal infections are an emerging but underrecognized concern in diabetic foot ulcers (DFU); and may contribute to delaying healing and complicate clinical outcomes. This study reviewed available evidence to identify the neglected contribution of fungi to DFUs and to explore their antifungal susceptibility patterns.

**Methods:**

A systematic search in PubMed, Web of Science, and Google Scholar databases for articles published from 2021 to 2025. Eligible articles were screened, extracted, and reported according to PRISMA guidance, and methodological quality was assessed using JBI tools.

**Results:**

Of the 1,030 publications identified, 26 studies comprising 3,851 patients met the inclusion criteria, all reporting fungal involvement in DFUs. *Candida* spp. were the most commonly isolated organisms, with fluconazole resistance reaching up to 23%. Filamentous fungi accounted for 21.2% of reported cases, predominantly *Aspergillus niger* and *Aspergillus fumigatus*; *Rhodotorula mucilaginosa* and *Cutaneotrichosporon debeurmannianum* were also identified.

**Conclusions:**

These findings suggest a potentially important role of fungi in DFUs; however, evidence on antifungal susceptibility remains limited. Therefore, greater attention to fungal involvement is warranted to reduce infection burden, minimize complications, and improve health outcomes and quality of life in patients with diabetes.

**Systematic review registration:**

https://www.crd.york.ac.uk/PROSPERO/view/CRD420261404556, identifier CRD420261404556.

## 1 Introduction

Diabetic foot ulcers (DFU) are the most common types of chronic wounds and a common complication of diabetes, and can lead to a significant social, psychological, and economic burden on both patients and the health sector (Graves et al., 2022; Ge and Wang, 2023). Annually, there are 9.1–26.1 million cases of DFU worldwide, with a prevalence rate of 19–35% (Falanga et al., 2022; Armstrong et al., 2023). In the upcoming years, it is expected that the incidence of DFU will increase in parallel with the growing prevalence of diabetes. These ulcers account for around 20% of lower-extremity amputations, with recurrence rates of up to 65% in 3 to 5 years and mortality rates ranging from 50% to 70% (Meloni et al., 2020; Macdonald et al., 2021). DFU management is not only clinically challenging but also imposes a high economic burden, with costs reaching up to $30,000 for major amputations and significantly impacting healthcare systems (Driver et al., 2010).

**Figure 1:**
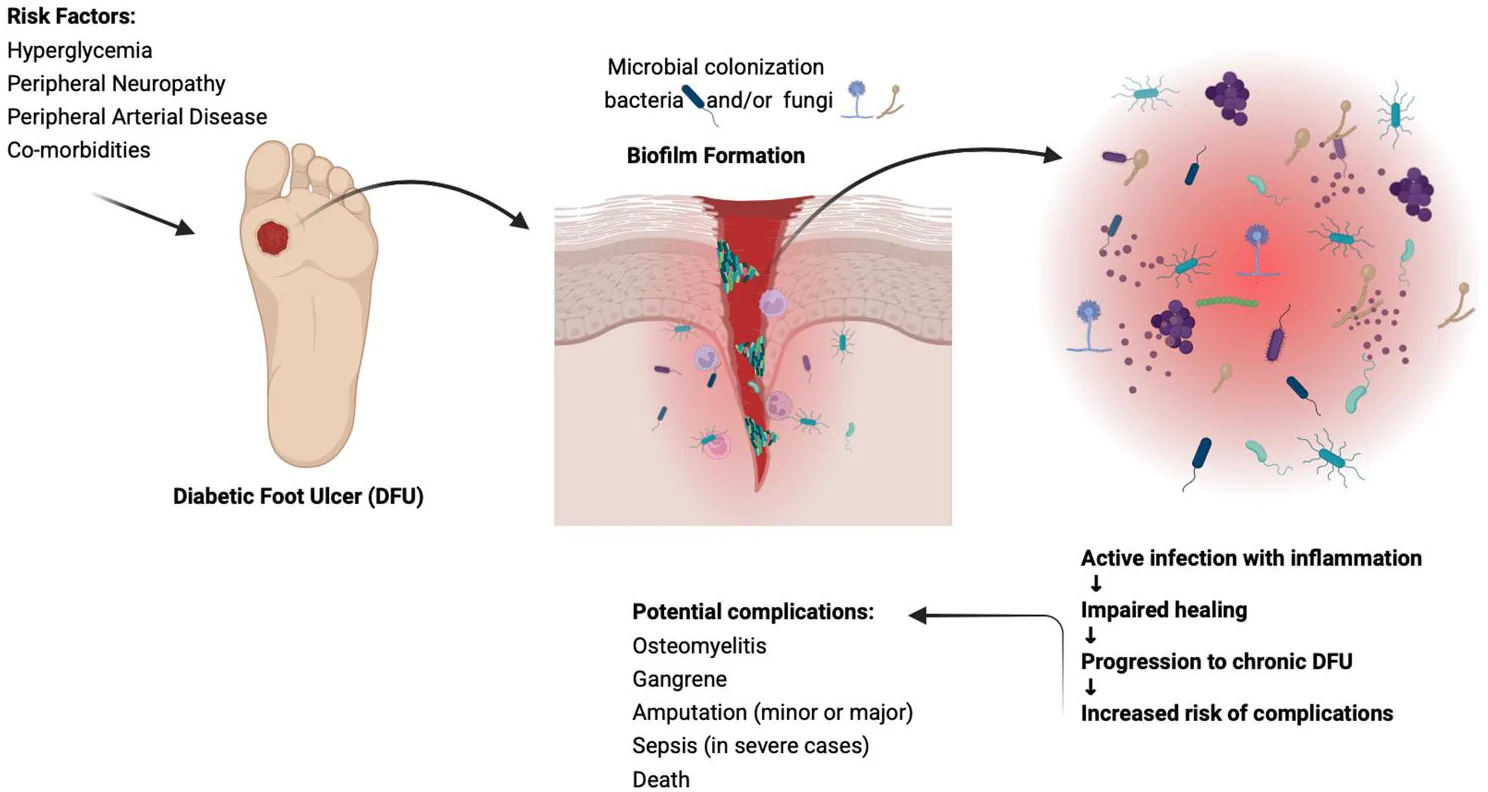
Pathophysiology and microbial progression in diabetic foot ulcers. Hyperglycemia and associated comorbidities predispose patients to diabetic foot ulcers, which serve as a niche for bacterial and fungal colonization. This microbial burden can delay healing and lead to severe complications such as gangrene and amputation. The illustration was created using BioRender (https://biorender.com).

Diabetic ulcers are highly susceptible and predisposed to invasion by many pathogenic and opportunistic microorganisms, including bacteria and fungi that mainly contribute to continued inflammation and delayed healing (**Figure 1**) (Uberoi et al., 2024). Diabetic foot infection (DFI) affects approximately 60% of DFU and confers substantial clinical consequences (Lavery et al., 2020; Macdonald et al., 2021; Chen et al., 2023). Therefore, DFIs represent a significant clinical challenge that requires an understanding of the microbiological nature of these infections. However, effective management primarily depends on the early detection of the causative agent, particularly since the skin harbors diverse microbiota, including both bacteria and fungi. Distinguishing between microbial colonization and true infection is crucial for the appropriate management of DFU. Various microorganisms can colonize ulcers without causing tissue damage or triggering the immune system. In contrast, infections are caused by microbial invasion and activate a host immune response (Sachdeva et al., 2022; Short et al., 2023). The transition from colonization to infection is a dynamic process, driven by a combination of the host’s immune status and the characteristics of bioburden, such as a shift to more pathogenic species with their ability to form biofilm and increase microbial load (Pouget et al., 2020; Sachdeva et al., 2022).

Several previous studies have demonstrated that DFUs are polymicrobial in nature (Heravi et al., 2019; Sachdeva et al., 2022; Qu et al., 2024; Allkja et al., 2025). However, most investigations based on conventional microbiological culture have primarily focused on bacterial involvement. For instance, a large systematic review including 11483 patients and 12292 strains of pathogenic organisms in China showed the microbial spectrum isolated from foot ulcers among diabetic patients was overwhelmingly bacterial, comprising 52.4% of Gram-negative and 43.4% of Gram-positive bacteria, whereas fungi represent only 3.7% of isolates. Among the isolated fungi, *Candida albicans* was the most abundant, accounting for about 55.6% (Du et al., 2022). Despite the scale of this analysis, fungal diversity and its clinical relevance were not comprehensively explored, suggesting that fungal involvement in DFUs remains underrepresented in large epidemiological studies, despite the recognized polymicrobial nature of these wounds. In addition, filamentous fungi and yeasts have been detected to cause DFI by various studies (Chellan et al., 2010; Fata et al., 2011; Sanniyasi et al., 2015; Kareliya et al., 2019; Ahmed et al., 2021). *Candida* spp. is determined to be the main agent involved in DFI (Chellan et al., 2010; Fata et al., 2011; Sanniyasi et al., 2015; Kareliya et al., 2019; Ahmed et al., 2021). It has been reported that 23% of DFUs were positive for fungal species (Dowd et al., 2008; Ge and Wang, 2023). Investigation conducted in Mumbai found that, among 41 patients who underwent lower limb amputation, 70.73% had fungal infection; the majority of them were *C. albicans* 40%, followed by *Penisillium Marneffei* (15%), *Pichia kudriavzevii (C. krusei)*, *Cladosporium carriani* and *Aspergillus niger* 10% for each. Both *Nakaseomyces glabratus (C. glabrata)* and *Fusarium* were each identified in 7.5% of cases (Kareliya et al., 2019). Another investigation in India was able to detect fungal species (19%) in DFU, where 68.42% co-existed with bacterial species in some cases, and 31.58% of them were isolated growths that were not associated with any bacteria. The most common isolated fungi being *candida* 26.32%, followed by *Aspergillus* spp. 21.05% (two isolates of *Aspergillus niger* and one each of *A. fumigatus* and *A. flavus*); followed by *Fusarium* spp. and *Trichophyton* spp. (15.79% each), *Penicillium* spp. (10.53%), and single isolates of *Acremonium* and *Trichosporon* (5.26% each) (Raza and Anurshetru, 2017).

A large meta-analysis included 112 studies, and 22198 microbial isolates showed that bacteria are the predominant cause of DFI; however, 258 fungal isolates were also identified (Macdonald et al., 2021). highlighting the often-overlooked role of fungi in DFU. The contribution of fungal infections to DFU has been understudied and scarcely investigated, with the majority of previous studies emphasizing bacterial etiologies. Although bacteria are widely recognized as the predominant cause of DFI, the contribution of fungi has been largely overlooked. Previous studies have primarily focused on bacterial pathogens, with limited investigation into fungal involvement and the complex microbial interactions that may influence the pathogenesis and clinical outcomes of DFUs. In addition, the skin harbors a diverse microbiota composed of bacteria and fungi in both diabetic and non-diabetic individuals (Erin Chen et al., 2018; Harris-Tryon and Grice, 2022; Sachdeva et al., 2022), making the scarcity of studies on fungal participation in diabetic ulcers a notable gap in current research.

Significantly, the scarcity of studies on fungal involvement in diabetic foot ulcers represents a notable gap in current research. In routine DFU management, which traditionally focuses on bacterial pathogens as the primary cause of infection, and fungal involvement is considered less common, and not all fungal isolates represent true infection, fungal diagnostics are often not performed unless specifically requested. This limited incorporation may contribute to the underrecognition of fungal infections, particularly in chronic or non-healing ulcers, potentially impacting treatment outcomes and antimicrobial stewardship, and may also contribute to the development of antimicrobial resistance. This study provides a comprehensive synthesis of the current literature to highlight the emerging and often overlooked role of fungi in diabetic foot ulcers. Furthermore, it discusses the diagnostic landscape for detecting these fungal pathogens and outlines key areas for future research, particularly in regions with limited data.

## 2 Methods

### 2.1 Study design and article search strategies

This systematic review was prospectively registered in the International Prospective Register of Systematic Reviews (PROSPERO) under registration number CRD420261404556.The literature search for this systematic review was conducted on 23 September 2025 according to the Preferred Reporting Items for systematic review and meta-analysis (PRISMA) guidelines. A systematic search was conducted in PubMed, Web of Science, and Google Scholar databases supplemented by manual reference screening and citation tracking to identify all relevant studies in the last five years. The literature search was restricted to studies published between 2021 and 2025 to provide an up-to-date synthesis of recent evidence of fungal involvement in DFUs, particularly in light of advances in fungal diagnostic methods and antifungal susceptibility testing. Furthermore, the microbiology of DFUs has been extensively reviewed elsewhere; thus, this review was intended to update the evidence base with a specific focus on recent developments in fungal epidemiology, diagnostic approaches, and antifungal susceptibility. Combinations of search terms used (“diabetic foot ulcer” OR “diabetic foot infection”) AND (fungi OR fungal OR mycosis OR Candida OR Aspergillus). The complete electronic search strategy for each database is provided in (**Supplementary Table 1**) in accordance with the PRISMA 2020 recommendations. After screening titles and abstracts for relevance, full-text articles were assessed for eligibility based on the following criteria: (1) Patients with clinically confirmed DFU were the study participants, (2) studies published between 2021 and 2025, (3) original research studies, (4) studies using microbiological and/or molecular methods for pathogen identification, and (5) publication in English. Review articles and studies focusing exclusively on bacteria, with no assessment of fungal involvement in DFUs, were excluded. For this review, the term “fungal isolation” refers to the detection of fungal organisms from a DFU sample in the absence of confirmed invasive infection, whereas “fungal infection” is used only where the included studies explicitly reported in clinical signs of infection or histopathological confirmation of tissue invasion. Where included studies did not distinguish between these two states, this ambiguity is noted explicitly throughout the study. Furthermore, because the included studies employed diagnostic methods with varying sensitivity and specificity, a study was considered to report a fungal-positive-DFU if fungal organisms were detected using the diagnostic method applied in the original study. This operational definition was adopted to facilitate synthesis across a methodologically heterogeneous evidence base. However, it should be recognized that fungal-positive classifications reflect varying levels of diagnostic certainty rather than a uniform diagnostic threshold across all included studies.

### 2.2 Data extraction and quality assessment of included studies

Data were extracted using a pre-designed Excel spreadsheet. The data extraction tool included the following variables: authors, year of publication, country, study design, number of patients, number of isolates, type and number of fungal and bacterial species, antifungal susceptibility testing (AFST), antifungal agents, and laboratory methods used for detection. The methodological quality of the included studies was assessed using the Joanna Briggs Institute (JBI) critical appraisal tools, with checklists selected according to each study design (Moola et al., 2015). Specifically, the JBI analytical cross-sectional checklist was used for cross-sectional studies, while the JBI checklist for case reports was applied to case-based studies. Articles were reviewed through title, abstract, and full-text screening by two independent reviewers with disagreements resolved by consensus or referral to a third reviewer.

### 2.3 Data analysis

A quantitative meta-analysis was not performed because of substantial methodological and clinical heterogeneity across the included studies, including differences in study design, diagnostic methodologies, outcome definitions (fungal isolation versus confirmed infection), and specimen collection techniques. Furthermore, prevalence estimates were reported using inconsistent denominators, with some studies reporting findings based on the total number of patients, others on total isolates, and others on fungal-positive samples, making direct statistical pooling inappropriate. The inclusion of case reports alongside observational studies further limited the feasibility of quantitative synthesis because of the absence of a common comparator population. Therefore, a descriptive synthesis was considered the most appropriate and transparent approach for summarizing the available evidence, consistent with current methodological guidance for systematic reviews of prevalence data. All statistical analyses were performed using descriptive statistics with R software (version 4.5.0). The findings are presented in tables and graphical representations.

## 3 Results

### 3.1 Study characteristics

**Figure 2:**
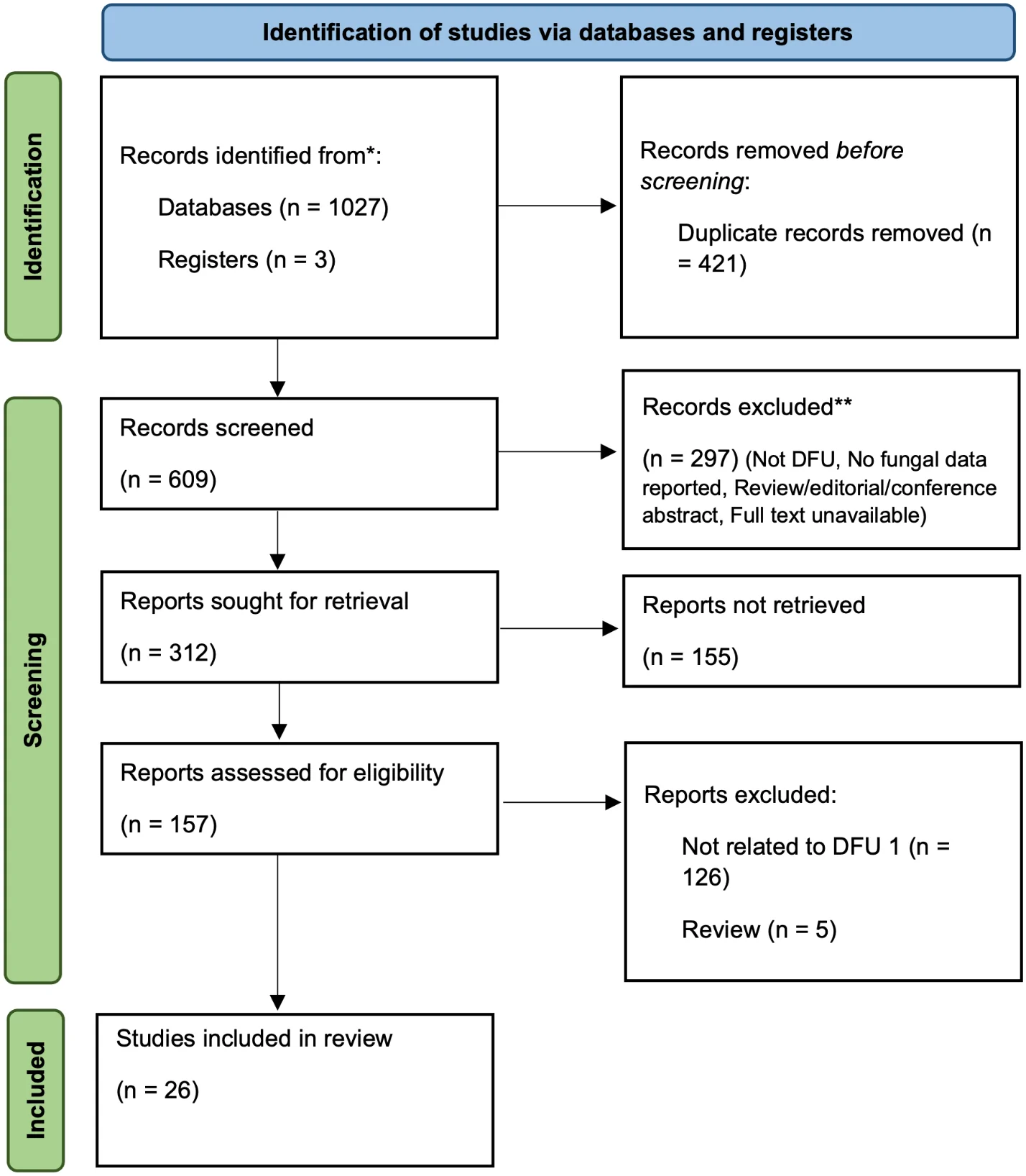
PRISMA flow diagram for the literature search and study selection. As per the PRISMA 2020 standards, 1,027 records were identified through a search across PubMed, Web of Science, and Google Scholar, along with 3 additional records identified through reference list screening. After removing 421 duplicate records, 609 records were screened, 157 full-text articles were assessed for eligibility, and 26 studies were included after applying the inclusion and exclusion criteria. The diagram was created using the PRISMA Flow Diagram tool available at PRISMA Flow Diagram – ESTECH (https://estech.shinyapps.io/prisma_flowdiagram/).

The database search across PubMed, Web of Science, and Google Scholar identified 1027 records, and three additional articles were retrieved through reference list screening. After removing 421 duplicates, 609 articles remained for title and abstract screening. Of these, 297 records were excluded, leaving 312 reports sought for retrieval; 155 could not be retrieved, and the remaining 157 were assessed for eligibility. Of these, 131 reports were excluded (126 not related to DFU; 5 reviews), resulting in 26 studies meeting the final inclusion criteria for analysis. The study selection process is summarized in the PRISMA flow diagram (**Figure 2**).

**Figure 3:**
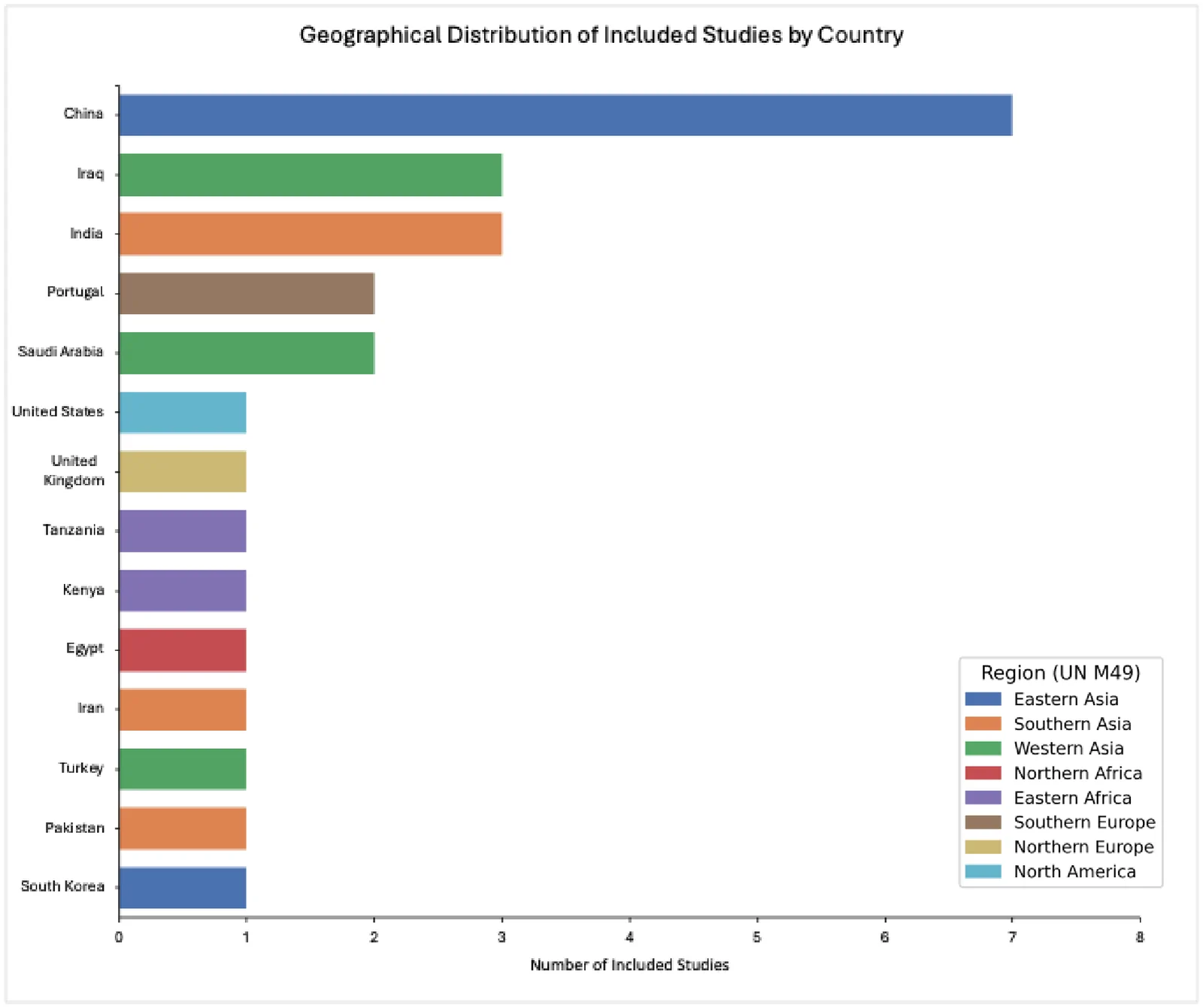
Geographical distribution of included studies by country. The bar chart illustrates the number of included studies (n = 26) conducted in each country, ranked in descending order and color-coded by region according to the United Nations M49 geoscheme. China contributed the largest number of studies (n = 7, Eastern Asia), followed by India and Iraq (n = 3 each), and Saudi Arabia (n = 2); the remaining ten countries each contributed a single study, covering Western Asia, Southern Asia, Eastern Africa, Northern Africa, Northern Europe, Southern Europe, and North America.

A total of 26 studies were included in this review. Among these, the majority were prospective studies 14 (53.8%), followed by retrospective studies 9 (34.6%), while case studies accounted for 3 (11.5%) (**Table 1**). The three included case reports were evaluated descriptively and interpreted independently of the observational research due to their differing methodological design and clinical purposes. Geographically, the studies were widely distributed, with the highest contribution from China (n = 7), followed by India and Iraq (n = 3 for each) and Saudi Arabia, and Portugal (n = 2 each). Other countries, including Kenya, Iran, the United Kingdom, Tanzania, Turkey, Egypt, the United States, Pakistan, and South Korea, each contributed one study (**Figure 3**; **Table 1**).

**Table 1:** Clinical characteristics of patients included in the studies.

Study number. (author, year)	Study country	Study design	Sample size	Type of diabetes (T1DM/T2DM) ^†^	Gender (male/female)	Mean age (years)	HbA1c (%) ^†^	Duration of DM (years)	Study period
1. (João et al., 2021)	Portugal	Case study	1	100%/0	100%/0	65	NM	16	NM
2. (Saeb et al., 2021)	Saudi Arabia	Prospective study	38	NM	76.3%/23.7%	NM	9.8	19	2016-2017
3. (Chai et al., 2021)	China	Retrospective study	102	NM	63.7%/36.3%	72.42 ± 8.43	> 10.0	10.09 ± 3.40	2016–2019
4. (Li et al., 2022)	China	Retrospective study	101	97.0%/3.0%	66.3%/33.7%	60.32 ± 11.62	11.46 ± 2.79	10.27 ± 7.09	2013-2020
5. (Yoo et al., 2022)	South Korea	Case study	1	100%/0	100%/0	68	NM	NM	NM
6. (Musyoki et al., 2022)	Kenya	Prospective study	152	98%/2%	61.0%/39.0%	50.7 ± 12.9	7.10 ± 1.67^‡^	11	2018-2020
7. (Kandregula et al., 2022)	India	Prospective study	60	NM	83.3%/16.7%	57.63 ± 11.31	10.56 ± 2.81	7.89 ± 6.14	3 months
8. (Moslemi et al., 2023)	Iran	Prospective study	100	95%/5%	84.6%/15.4%	62.1 ± 10.8	9.4 ± 1.8	>10	2019-2020
9. (Liu et al., 2023)	China	Retrospective study	155	NM	NM	NM	NM	NM	NM
10. (Devasia et al., 2022)	India	Prospective study	66	NM	89.0%/11.0%	NM	9.68	NM	NM
11. (Shalaby et al., 2023)	Egypt	Prospective study	100	NM	61.0%/39.0%	56.8	NM	13.7	2020-2021
12. (Helal et al., 2023)	Iraq	Prospective study	100	NM	54.0%/46.0%	56.9 ± 7.8	12.13	15	2021-2022
13. (Kibunto et al., 2023)	Tanzania	Prospective study	71	98.6%/1.4%	66.2%/33.8%	59.2 ± 13.0	NM	7	2022
14. (Shi et al., 2022)	China	Prospective study	89	NM	62.9%/37.1%	53.2 ± 5.4	7.9 ± 1.4	14.8 ± 2.9	2018-2021
15. (Liu et al., 2023)	China	Retrospective study	581	98.62%/1.20%	65.06%/34.94%	61.29 ± 11.5	NM	10.45 ± 6.84	2018-2021
16. (Azharuddin et al., 2023)	India	Prospective study	251	NM	76.5%/23.5%	54.1 ± 13.3	NM	11–15	2018-2020
17. (Coşkun et al., 2024)	Turkey	Retrospective study	262	NM	71.4%/28.6%	62.595 ± 11.489	NM	NM	2021-2022
18. (Frost et al., 2024)	United States	Case study	1	NM	0/100%	78	NM	NM	NM
19. (Alkhatieb et al., 2024)	Saudi Arabia	Retrospective study	95	96.9%/3.1%	67.7%/32.3%	63.03 ± 10.88	NM	NM	2019-2021
20. (Al-Chalabi et al., 2024)	Iraq	Prospective study	203	NM	51.7%/48.3%	56.50 ± 10.86	NM	10.96 ± 4.63	2021-2022
21. (Mustafa et al., 2024)	Iraq	Prospective study	250	NM	NM	NM	NM	NM	2023
22. (Xie et al., 2024)	China	Retrospective study	76	NM	NM	NM	NM	NM	2016-2020
23. (Gonçalves et al., 2024)	Portugal	Retrospective study	60	76.7%/15.0%/8.30% Others ^§^	61.7%/38.3%	59.1 ± 11.5	9.21 ± 0.09	22.5 ± 11.9	2020-2023
24. (Allkja et al., 2025)	United Kingdom	Prospective study	128	NM	NM	NM	NM	NM	NM
25. (Zhang et al., 2025)	China	Retrospective study	697	NM	63.4%/36.6%	63.67 ± 11.72	> 9	11.14 ± 7.30	2016-2023
26. (Kainat et al., 2025)	Pakistan	Prospective study	111	NM	76.0%/24.0%	54 ± 10	> 7.0	NM	2021

^†^% as reported in article; NM, Not Mentioned; T2DM, Type 2 Diabetes Mellitus; T1DM, Type 1 Diabetes Mellitus; HbA1C, Hemoglobin A1C.

^‡^The value represents random blood glucose levels and not HbA1c. Mean and SD were estimated from the median and IQR, assuming a normal distribution.

^§^Others DM (Post-transplant diabetes mellitus, MODY, steroid-induced diabetes).

### 3.2 Clinical characteristics of patients included in the studies

The 26 studies collectively included 3851 patients, with a mean age of 61 years (range from 51 to 78 years). Most patients had type 2 diabetes mellitus (T2DM) (96.8%), while type 1 diabetes mellitus (T1DM) accounted for only 2.7% of cases. Other types of diabetes, including steroid-induced diabetes mellitus (SIDM), post-transplant diabetes mellitus (PTDM), and Maturity-onset diabetes of the young (MODY), were rarely reported (0.4%). Several studies did not specify diabetes type and were excluded. The weighted mean HbA1c levels across studies reporting glycemic data (n = 1676 patients) were approximately 9.6%, ranging from 7.0% to 12.2%. Several studies did not report HbA1c values and were excluded from the analysis. The weighted mean duration of diabetes mellitus among included patients was approximately 11.6 years, with values ranging from 7 to 23 years. Across the included studies, male patients were consistently predominant, with proportions ranging from 51.7% to 89%, while female proportions ranged from 11% to 48.3%. In the pooled analysis (n = 3242), males accounted for 66.5% of cases compared to 33.9% females (**Table 2**).

**Table 2:** Distribution and relative frequencies of yeast isolates.

Species	n	%
***Candida albicans***	**19**	**28.8%**
**Non-*albicans Candida* (total)**	**44**	**66.7%**
*C. parapsilosis*	12	18.2%
*C. tropicalis*	10	15.2%
*Nakaseomyces glabratus (C. glabrata)*	7	10.6%
*Pichia kudriavzevii (C. krusei)*	4	6.1%
*C. guilliermondii*	3	4.6%
*C. lusitaniae*	3	4.6%
*C. famata*	2	3.0%
*C. dubliniensis*	2	3.0%
*C. auris*	1	1.5%
***Trichosporon* spp.**	1	1.5%
***Cutaneotrichosporon debeurmannianum***	1	1.5%
***Rhodotorula mucilaginosa***	1	1.5%
**Total yeasts** ^†^	**66**	**100**

^†^Total reflects all yeast isolates identified to at least the genus level with a reported numerical count (n = 66); studies reporting *Candida* spp. without species-level identification were excluded from this denominator.

^N.B^Includes two isolates derived from single-patient case reports (*Trichosporon* spp.*, C. debeurmannianum)*.

Bold values indicate main categories, aggregate groups, or taxa reported separately from the Candida categories.

### 3.3 Microbiological pattern of fungal and bacterial isolates

Fungal organisms were consistently reported across all included studies. Most included studies reported polymicrobial infections involving both bacterial and fungal pathogens (n = 18, 69.2%); however, these studies did not consistently distinguish between active infection and colonization. Monomicrobial fungal infections were reported in 2 studies (7.7%), which were case reports. In addition, 23.1% of included studies did not specify infection types. Regarding fungal infection, yeasts constituted the majority of fungal isolates (72.3%), followed by molds (21.3%) and dermatophytes (6.4%). *Candida* species were the dominant yeasts, with *C. albicans* being the most frequently reported species (28.8%), while non-albicans *Candida* accounted for about 66.7% of reported yeast isolates. The most frequently reported non-albicans species included *C. parapsilosis* (18.2%) and *C. tropicalis* (15.6%), followed by *N. glabratus* (10.6%). Less commonly reported species included *Pichia kudriavzevii (C. krusei)* (6.1%), *C. guilliermondii* and *C. lusitaniae* (4.6% each), while *C. famata* and *C. dubliniensis* were identified at lower frequencies (3.0% each), and *C. auris* was reported in (1.5%) of yeast isolates in the included studies. In addition, non-*Candida* yeasts such as *Trichosporon* spp. were reported in (1.5%). Very rare clinically reported yeast and yeast-like fungi, *Rhodotorula mucilaginosa* and *Cutaneotrichosporon debeurmannianum, were* reported in equal proportion (1.5% for) (**Table 2**).

Among molds, *Aspergillus* spp. was the most reported (60.0%), followed by *Penicillium* spp. (15.0%) and *Fusarium* spp. (10.0%). The species-level identification was available for most reported isolates. Significantly, some studies reported only genus-level identification, which was analyzed separately to avoid misclassification bias. Among *Aspergillus* spp., *A. niger* was the most frequently reported (20.0%), followed by *A. fumigatus* (15.0%), *A. flavus* (10.0%), and *A. nidulans*, accounting for (5%) of isolates. While other filamentous fungi, including *Alternaria* spp., *Cladosporium* spp., and *Rhizopus* spp. accounted 15.0% (**Table 3**). Dermatophytes were less frequently reported, mainly represented by *Trichophyton* spp. (66.7%) and *Microsporum* spp. (33.3%) respectively (**Table 4**).

**Table 3:** Distribution and relative frequencies of filamentous fungi isolates.

Group	Organism	n	%
***Aspergillus* spp.**	*Aspergillus* spp. ^†^	2	10.0%
	*Aspergillus niger*	4	20.0%
	*Aspergillus fumigatus*	3	15.0%
	*Aspergillus flavus*	2	10.0%
	*Aspergillus nidulans*	1	5.0%
	**Total**	**12**	**60**.0**%**
***Penicillium* spp.**	*Penicillium* spp.	3	15.0%
	**Total**	**3**	**15**.0**%**
***Fusarium* spp.**	*Fusarium oxysporum*	1	5.0%
	*Fusarium* spp. ^†^	1	5.0%
	**Total**	**2**	**10**.0**%**
**Other molds**	*Alternaria* spp.	1	5.0%
	*Cladosporium* spp.	1	5.0%
	*Rhizopus* spp.	1	5.0%
	**Total**	**3**	**15**.0**%**
**Overall Filamentous fungi**		**20**	**100**.0**%**

^†^*Aspergillus* spp. and *Fusarium* spp. indicate isolates identified only at the genus level without species-level differentiation.

The percentages were calculated based on the total number of filamentous fungal isolates reported across all included studies (n = 20).

N.B. Includes *Fusarium oxysporum* derived from single-patient case reports.

The bold values indicate the total number and relative frequency of isolates within each fungal group.

**Table 4:** Distribution and relative frequencies of dermatophyte isolates.

Group	Organism	n	%
***Trichophyton* spp.**	*Trichophyton* spp. ^†^	1	16.7%
	*Trichophyton mentagrophytes*	2	33.3%
	*Trichophyton interdigitale*	1	16.7%
	**Total**	**4**	**66.7%**
***Microsporum* spp.**	*Microsporum* spp. ^†^	1	16.7%
	*Microsporum canis*	1	16.7%
	**Total**	**2**	**33.3%**
**Overall Dermatophytes**		**6**	**100**.0**%**

^†^*Trichophyton* spp. and *Microsporum* spp. indicate isolates identified at the genus level only, without species-level differentiation, likely due to the absence of molecular identification methods.

Percentages are calculated relative to the total number of dermatophyte isolates reported across all included studies (n = 6).

The bold values indicate the total number and relative frequency of isolates within each fungal group.

### 3.4 Antifungal susceptibility patterns

AFST was performed in a limited number of studies 3 (11.5%), while the majority of studies 23 (88.5%) did not report standardized susceptibility testing methods. Among those not performing AFST, three studies described antifungal use patterns, predominantly highlighting voriconazole as a commonly used therapeutic agent. Among the studies that performed antifungal susceptibility testing, commercial automated systems such as Sensititre™ YeastOne and VITEK^®^ 2 were used. One study reported the use of a proprietary automated system without further methodological details (**Table 5**). Overall, *Candida* spp. demonstrated high susceptibility to commonly used antifungal agents, particularly azoles and amphotericin B, with reported susceptibility rates ranging from 77% to 97%. Flucytosine and amphotericin B exhibited the highest antifungal activity, whereas echinocandins—especially caspofungin—showed relatively higher resistance rates. Despite limited AFST data, species-specific differences in antifungal susceptibility were observed. *C. tropicalis* demonstrated the highest resistance rates, including fluconazole resistance of up to 23.0%, as well as resistance to amphotericin B and voriconazole (7.7% each). In contrast, *C. albicans* remained largely susceptible, with a resistance rate of approximately 13.3%. Additionally, *Trichosporon* spp. exhibited intrinsic resistance to echinocandins despite susceptibility to azoles (**Table 5**). To facilitate these findings for clinical interpretation, antifungal susceptibility data from the included studies are summarized descriptively according to fungal species and antifungal agent (**Table 6**).

**Table 5:** Microbiological characteristics of isolates and laboratory diagnostic methods used in identification.

Study number. (author, year)	Total isolates (bacteria/fungi)	Fungal species identified	Bacterial species identified	Antifungal susceptibility testing (AFST)/Antifungal susceptibility pattern	Laboratory identification methods
1. (João et al., 2021)	2 (1/1)	*Fusarium oxysporum* (1/1; 100%)	*Pseudomonas* (MDRO) was co-isolated in 100%	NM Voriconazole and systemic antibiotherapy	**Histological analyses** · Hematoxylin and eosin (HE) stain · Grocott’s stain
2. (Saeb et al., 2021)	176	**By traditional culture** **Patient DFU21:** *Candida glabrata* (100%) & No bacteria detected **Patient DFU37:** *C, albicans* (50%) & *Entrococcus cloa*cae (50%).	**DFU21:** 16S revealed Staphylococcus spp.	NM	**Traditional culture** for bacterial and fungal growth. Culture-enriched microbiome **16S rRNA sequencing**
3. (Chai et al., 2021)	NM	**Fungi (2.0%) including:** *C. albicans* 2%	**Gram-negative bacteria** (54.9%) **Gram-positive bacteria (43.1%)**	NM	**VITEK- 60**
4. (Li et al., 2022)	124 (118/6)	**Fungi (4.8%)** *N. glabratus* (2.4%), *C. albicans, C. parapsilosis, C. tropicalis* (0.8% for each) *N. glabratus* was cultured together with *Klebsiella pneumoniae*	**Gram-positive bacteria (61.3%)** **Gram-negative bacteria (33.9%)**	NM	**VITEK-MS mass spectrometer** **VITEK2-Compact**
5. (Yoo et al., 2022)	1	*Cutaneotrichosporon debeurmannianum* (1/;, 100%)	Not detected	**Sensititre™ YeastOne (SYO) plate.** · Highly susceptible to azoles and amphotericin B · Completely resistant to all echinocandins	**MicroIDSys MALDI-TOF MS system** **Molecular sequencing** ITS1/ITS2 & D1/D2
6. (Musyoki et al., 2022)	102 (59/43)	***Candida* species** (n=35): *C. albicans (75%)* *C. lusitaniae (8%)* *C. dubliniensis (5%)* *C. dubliniensis (5%)* *C. parapsilosis (2%)* *C. tropicalis (2%)* *N. glabratus (2%)* *C. famata (2%)* ***Moulds (n=8):*** ***Penicillium* spp.** *(38%)* *Aspergillus* spp. (25%) *Microsporum* spp. (25%) *Trichophyton mentagrophytes (12%)*	Bacterial findings (n = 59): · Gram negative bacili (51%) · Gram positive cocci clusters (15%) · Mixed bacterial infection of both (34%)	**VITEK^®^ 2 System (AST-YS08):** *Candida* spp. high susceptibility (77–97%) to voriconazole, flucytosine, micafungin, caspofungin, amphotericin B, and fluconazole, and **highest resistance to Caspofungin** · *C. albicans* exhibited 81–96% susceptibility to amphotericin B and flucytosine, while 26% resistance to **caspofungin,** · Non-*albicans* demonstrated 90–100% susceptibility to most of the tested antifungal agents.	**Microscopic Examination 10% KOH** **Culture:** SDA with chloramphenicol. **VITEK - 2 System** **MALDI-ToF MS**
7. (Kandregula et al., 2022)	NM	**The prevalence of fungal infection was 31.7%:** ***Candida***: *C. albicans (33.33%)* *C. tropicalis (23.81%)* *C. parapsilosis (19.04%)* *C. lusitaniae* *N. glabratus* **Filamentous fungi:** *Altenaria* spp. *(4.76%)* *Penicillium* spp. *(4.76%)* *Trichophyton* spp. *(4.76%)*	NM	NM	**Microscopic Examination** 10% KOH and Gram-stain **Culture**: SDA with chloramphenicol. Lactophenol Cotton Blue (LPCB) Slide culture technique
8. (Moslemi et al., 2023)	NM (/5)	***Candida* (20%) including:** *C. parapsilosis (71.5%)* *C. albicans (14.3%)* **Filamentous fungi were not detected**		NM	**Microscopic examination:** 10% KOH- LPCB **Culture**: SDA with chloramphenicol SDA with chloramphenicol and cycloheximide CHROMagar *Candida* **Mount Slide Culture Technique** **PCR** amplify the ITS1–5.8S–ITS4 rDNA
9. (Liu et al., 2023)	162 (156/6)	6 Strains fungi (species NM)	73 strains Gram-positive bacteria, 83 strains Gram-negative bacteria.	NM Fungi were more sensitive to **voriconazole** and **itraconazole**	NM
10. (Devasia et al., 2022)	NM	*C. tropicalis* (100%) Molds were not isolated	*S. aureus* isolates was more (61%), followed by *Pseudomonas* spp. (29%), *Proteus* spp. (7%) and *Klebsiella* spp. (3%).	NM	**Culture:** slants SDA ·**Direct microscopic examination:** KOH (10%) wet mount & Gram stain – initial fungal detection · Filamentous fungi – slide culture on PDA + LPCB staining · Yeasts – species identification using **HiCrome™ *Candida* differential agar**
11. (Shalaby et al., 2023)	143 (126/17)	***Candida* spp.** *C. albicans* (71.4%) *C. tropicalis* (21.4%) *N. glabratus* (7.1%) ***Proteus* spp. was the most common associated with fungi (47%) & *Pseudomonas* spp.**	Most common bacteria: *Proteus* species (37.3%) and *Pseudomonas* spp. (30.16%).	NM	**Direct microscopic examination** (10% KOH **Culture:** SDA with chloramphenicol *Candida* on Chrom agar · VITEK 2 compact system **PCR:** 18s rRNA gene
12. (Helal et al., 2023)	NM	**skin mycosis:** *Aspergillus flavus* (18.8%), *C.albicans* (12.5%), *A. niger* (7.8%), *Trichophyton mentagrophytes* (7.8%), *T. interdigitale* (6.3%), *C. dubliniensis* (3.1%), and *Microsporum canis (*1.6%). **nails scrapings: dermatophytosis (50.1%) and yeast spp. (13.9%)** *A. niger* (27.8%), *T. interdigitale* (16.7%), *C. dubliniensis* (8.3%), *C. albicans* (5.6%), *M. canis* (2.8%), *T*. *mentagrophytes* (2.8%).	**NM**	NM	**Direct microscopic examination:** KOH % test **&** LLPCB staining **Culture:** on SDA **Biochemical examinations**
13. (Kibunto et al., 2023)	92 (70/22)	**Total fungi (23.9%):** **Yeasts (31.8% of fungi)** *C. albicans* (42.8%) *P. kudriavzevii* (28.6%) *C. parapsilosis* (28.6%) **Moulds (68.2% of fungi)** *A.fumigatus* (86.7%)	**Total bacterial (76.1%):**	NM	**Tissue culture**
14. (Shi et al., 2022)	153 (148/5)	Fungal isolates (3.3%): *Candida* spp. (3.4%) *& A. niger* (2.2%)	**Gram-positive bacteria (52.3%) Gram-negative bacteria (44.4%)**	NM	**Standard culture techniques**
15. (Liu et al., 2023)	656 (622/21)	**Fungi (3.20%):** *C. albicans (33.33%)* *C. tropicalis (33.33%)* *C. parapsilosis (19.05%)*	Gram-positive bacteria (58.99%) Gram-negative bacilli (35.82%)	NM	**VITEK 2**
16. (Azharuddin et al., 2023)	NM/48	**Fungi 23.3%**: · 2% pure fungi · 21.3% mixed with bacteria **Yeast:** *C. tropicalis (27.08%)*, *C. albicans (20.83%).* *C. parapsilosis (8.33%)* *C. auris (4.17%)* *P. kudriavzevii, N. glabratus, C. orthopsilosis, Trichosporon* spp. *(2.08%)* **Molds:** *Aspergillus* spp. *(10.41%)* *Fusarium* spp. *(10.41%)* *Rhizopus* spp. *(4.16%)* *Penicillium* spp. *(4.16%)* *Cladosporium* spp. *(2.08%)*	NM	**The proprietary system:** **·** *C. tropicalis*: 23% resistant to fluconazole and 7.69% were resistant to voriconazole and amphotericin B · *C. albicans*: 13.3% resistant to fluconazole	**MALDI-TOF MS** **Microscopic examination**
17. (Coşkun et al., 2024)	432 (427/5)	*Candida* spp. (1.2%): *C. albicans* (0.4%)	**Gram-negative bacteria (57.60%)** **Gram-positive bacteria (41.20%)**	NM	**VITEK-2**
18. (Frost et al., 2024)	1	*Trichosporon asahii* (1/1; 100%)	Not detected	NM **Focuses on antifungal use: Voriconazole**	**Bone culture** **Histological study:** Grocott methenamine silver special stain
19. (Alkhatieb et al., 2024)	NM	*Candida* spp. (2.1%)	**Gram-negative** (38.5%) Gram-positive (29.2%)	NM	**Tissue culturing**
20. (Al-Chalabi et al., 2024)	NM	Fungi (65%) (58.4%) of C*andida* spp.: (*C. tropicalis, P. kudriavzevii, C. parapsilosis, C. lusitaniae, C. guilliermondii*), *C. albicans* 13.3%	**NM**	NM	**Culture:** SDA &chromogenic agar **Germ tube formation test** **Phenotypic** **Microscopic examinations** **Vitek technique**
21. (Mustafa et al., 2024)	91 fungal isolates	**Yeasts (61.54%):** ***Candida* spp. (61.54%):** *C. albicans* (29.67%) *C. tropicalis* (24.18%) *P. kudriavzevii* (7.69%) ***Aspergillus* spp. (38.46%):** *A. fumigatus (19.78%)* *A. niger* ***(****14.29%)* *A. flavus (4.39%)*	**NM**	NM	**Direct microscopic examination:** KOH test & LPCB staining **Culture:** SDA Corn Meal Agar with Tween 80 (CMA) Chrome Agar (CCA) **PCR** ITS target
22. (Xie et al., 2024)	96 (92/4)	**Fungi 4.3%:** C. albicans (50%) *C. guilliermondii* (25%) ***A. fumigatus* (25%)**	Gram-negative bacilli 44.6% Gram-positive cocci 51.1%	NM	NM
23. (Gonçalves et al., 2024)	102 (98/4)	**Fungi (3.92%)** *C. parapsilosis* (2.94%) *C. albicans* (0.98%)	Gram-positive bacteria (60.8%) Gram-negative (31.4%)	NM	**MALDI-TOF MS**
24. (Allkja et al., 2025)	349 (NM/31)	*C. parapsilosis* (20/31, 64.5%) *C. albicans (7/31, 22.6%)* *N. glabratus (3/31, 9.7%)* *Rhodotorula mucilaginosa (1/31, 3.2%)*	**Gram negative** including: *Pseudomonas* spp., Enterics, and Coliforms	NM	**Culture:** Enhanced SDA + chloramphenicol, CHROMagar **MALDI-TOF** **ITS Real-Time qPCR Screening**
25. (Zhang et al., 2025)	891(873/18)	**Fungi** (2.0%): *Candida parapsilosis* (8/44.4%) *C.tropicalis* (4/22.2%) *C. guilliermondii* (3/16.7%) *C. albicans* (1/5.6%) *P. kudriavzevii* (1/5.6%) *C. famata* (1/5.6%)	**Gram-positive bacteria (47.0%)** **Gram-negative bacteria (51.0%)**	NM	**VITEK MS**
26. (Kainat et al., 2025)	NM(/29)	***Candida* spp. (62%):** *C. albicans (41.3%)* *C. tropicalis (13.7%)* *N. glabratus (6.9%)* *A. niger (38%)*	**(91%) bacterial growth**	NM	**BD Phoenix automated analyzer** **Biofilm formation assay (O’Toole method modified)**

% as reported in article; NM, Not Mentioned.

N.B. *N. glabratus* and *P. kudriavzevii* are the currently accepted names for *C. glabrata* and *C. krusei*, respectively.

Bold formatting is used to distinguish the main microbiological categories and key laboratory diagnostic information from the detailed findings reported within each category.

**Table 6:** Descriptive summary of antifungal susceptibility patterns by fungal species and antifungal drug class.

Source study	Fungal species	Antifungal agent drug class			
		Azoles	Polyenes (Amphotericin B)	Pyrimidine analogues (Flucytosine)	Echinocandins
(Yoo et al., 2022)	*C. debeurmannianum*	Susceptible	Susceptible	Not reported	Completely resistant
(Musyoki et al., 2022)	*Candida* spp. (pooled)	77–97% susceptible (voriconazole, fluconazole)	77–97% susceptible	77–97% susceptible	Highest resistance among agents tested
(Musyoki et al., 2022; Azharuddin et al., 2023)	*C. albicans*	13.3% resistant to fluconazole; voriconazole not separately reported	81–96% susceptible	81–96% susceptible	26% resistant to caspofungin
(Musyoki et al., 2022)	Non-*albicans Candida* (pooled, excluding *C. tropicalis*)	90–100% susceptible to most agents	90–100% susceptible	90–100% susceptible	90–100% susceptible
(Azharuddin et al., 2023)	*C. tropicalis*	23% resistant to fluconazole; 7.69% resistant to voriconazole	7.69% resistant	Not reported	Not reported

### 3.5 Laboratory identification methods for fungi in DFUs

A total of 25 out of 26 included studies demonstrated heterogeneity in the laboratory approaches used for fungal identification in diabetic foot-related infections. Culture-based methods, including Sabouraud dextrose agar (SDA), Sabouraud Cycloheximide Chloramphenicol Agar, and chromogenic media, were the most frequently applied diagnostic approach (n = 13). Similarly, advanced identification approaches such as matrix-assisted laser desorption/ionization time-of-flight mass spectrometry (MALDI-TOF MS), VITEK automated systems, and BD Phoenix automated analyzer were reported in an equal number of studies (n = 13). Direct microscopic examination using potassium hydroxide (KOH) preparation and Lactophenol Cotton Blue (LPCB) was applied in 9 studies. Molecular-based methods, including PCR amplification and ITS rRNA sequencing, were less frequently utilized (n = 5). Histopathological analysis, including hematoxylin and eosin and Grocott methenamine silver staining, was the least commonly employed method in two case-based studies (**Table 5**). Taken together, the concentration of evidence in a small number of countries and the predominant reliance on culture-based rather than molecular diagnostic methods suggest that current estimates of fungal prevalence and species diversity in DFUs may not be generalizable across regions or diagnostic settings. Future studies employing molecular identification techniques in underrepresented regions, particularly the Middle East and Africa, are needed to clarify whether the species distribution reported here reflects true geographical variation or differences in diagnostic sensitivity.

### 3.6 Quality assessment of included studies

The methodological quality of the included studies was assessed using the JBI Critical Appraisal Tool for Analytical Studies. A total of 26 studies were included; two of the three case reports achieved a score of 7 out of 8, while one achieved a score of six out of 8. The quality scores for the cross-sectional studies (n = 23) varied from 6 to 8. In particular, four studies had a score of 6 out of 8, nine received a score of 7 out of 8, and ten achieved complete ratings of 8 out of 8. The detailed domain-specific JBI critical appraisal results for each included study are presented in **Supplementary Table 2**. The majority of the included studies were of moderate to high quality. Methodological limitations were concentrated in two domains: clarity of inclusion criteria (Q1), rated as unclear in 10 of 26 studies (38.5%), and appropriateness of statistical analysis (Q7), rated as inadequate or unclear in 6 of 26 studies (23.1%). In contrast, domains related to exposure measurement, outcome assessment, and the use of standard diagnostic criteria were adequately reported across all included studies.

## 4 Discussion

DFUs are a serious public health issue that impose significant medical, psychosocial, and socioeconomic burdens due to their chronic and polymicrobial nature. Unlike bacteria, the clinical importance of fungi in DFUs is often overlooked, particularly with the presence of an open wound, antibiotic use, and fungal colonization, which create favorable conditions for infection (Short et al., 2023). This suggests that fungi may contribute to, rather than merely accompany, infection and delayed wound healing, although the observational nature of the included studies precludes causal inference. Therefore, this study aimed to explore the underrecognized contribution of fungal pathogens in DFUs based on previously published literature to provide a comprehensive overview of the current state of knowledge regarding their role.

While the International Working Group on the Diabetic Foot (IWGDF) guidelines clearly recognize the importance of managing fungal infections as part of preventative care and pre-ulcerative risk reduction, this recognition is not reflected in the current evidence base (Monteiro-Soares et al., 2024). Notably, the updated 2023 IWGDF/IDSA guidelines on the diagnosis and treatment of diabetes-related foot infections recommend that clinicians determine the causative microorganism through tissue-based culture and, where appropriate, molecular identification techniques (Monteiro-Soares et al., 2024). However, these recommendations are framed broadly around microbial identification in general, without specific guidance on when fungal-targeted investigations should be pursued in chronic or non-healing ulcers. This absence of fungus-specific diagnostic recommendations may partly explain why fungal diagnostics remain inconsistently applied in routine clinical practice. This gap is not limited to infection-specific guidelines: the recently published 2025 global candidiasis guideline (ECMM/ISHAM/ASM) similarly recommends combining traditional diagnostic methods with contemporary molecular and biomarker-based methodologies; however, it does not specifically address chronic wound-specific situations, such as DFUs (Cornely et al., 2025). Collectively, these observations underscore an important opportunity for future guideline development — specifically, clearer, evidence-based guidance on the indications for highlighting the necessity of DFU-specific diagnostics, particularly in ulcers that fail to respond to standard antibiotic therapy.

The available evidence synthesized in this review shows that fungi, including yeasts, yeast-like fungi, molds, and dermatophytes, were consistently reported across all included studies, regardless of microbial classification or study design. Furthermore, fungal organisms were reported as a co-isolated or contributing organism in DFIs with deep tissue invasion in several studies, while nearly 70.0% of studies reported fungi alongside bacterial isolates. The remaining studies confirmed the presence of fungi but did not determine whether the fungal infection was monomicrobial or polymicrobial (with bacteria). Significantly, those investigations un clarify the fungal role, either colonizer or an infection, which highlights that fungal involvement is a constant feature in DFU microbiology and emphasizes the connection between outcomes and wound characteristics and clinical aspects.

In 72.3% of the included investigations, yeasts—both *Candida* and non-*Candida* spp.—were the most commonly isolated fungi. According to (**Table 2**), *C. albicans* was the most commonly reported species (28.8%), followed by *C. parapsilosis, C. tropicalis, and Nakaseomyces glabratus (C. glabrata)* (18.2%, 15.2, and 10.6%), respectively. This finding is consistent with the opportunistic nature of *Candida* spp., which are known to colonize compromised tissues, thrive in the hyperglycemic environment typical of diabetic patients, and are frequently implicated as a contributing pathogen in chronic wound infection (Ge and Wang, 2023). Even though *C. auris* was only reported 1.5% of the time (**Table 2**), Its detection is clinically noteworthy since it is becoming recognized as a multidrug-resistant pathogen with important clinical and epidemiological consequences. This demonstrated the increasing clinical relevance of *Candida* spp. important fungal pathogens, highlighting their possible danger in medical environments and persistent infections (**Table 7**) (World Health Organization, 2022; Rodrigues and Nosanchuk, 2023; Bhargava et al., 2025).

**Table 7:** Fungal species isolated from DFUs and their key characteristics.

Fungal group	Predominant species	Notes
Yeasts	*Candida parapsilosis*, *C. tropicalis*, *C. albicans*	*C. parapsilosis* is increasingly reported as the most common isolate in some regional studies.
Emerging Yeasts	*Candida auris*, *Nakaseomyces glabratus (C. glabrata)*	*C. auris* is noted for its high resistance to fluconazole and environmental persistence.
Molds	*Aspergillus flavus*, *Aspergillus niger*, *Fusarium* spp.	Often isolated in chronic, deep-tissue infections; may be considered contaminants unless invasive forms are seen.
Dermatophytes	*Trichophyton rubrum*	Frequently coexist with ulcers as tinea pedis or onychomycosis, acting as a potential entry point for deeper infection.

Furthermore, *A. niger* was the most commonly reported (20%) in DFUs; this is consistent with their biofilm-forming natural characteristics (**Table 2**) (Ge and Wang, 2023). Additionally, 6.38% of fungal isolates were dermatophytes, including *Trichophyton* spp. and *Microsporum* spp. (**Table 4**). Dermatophytes may represent superficial colonization or infection, but in compromised skin barriers, they may contribute to tissue breakdown and complicate clinical management, especially in immunocompromised and poor glycemic control (Saeb et al., 2021). Overall, the clinical profile of the patients in the included studies is a crucial factor that could account for the occurrence of fungi in DFUs. The majority of patients were elderly, with a mean age of 61, and had long-standing DM up to 22 years, and poor glycemic control. Chronic hyperglycemia and aging are known to impair immunity and increase susceptibility to infections, particularly fungal colonization (**Figure 1**).

Another important consideration is the potential underestimation of fungal diversity in studies relying predominantly on conventional culture-based identification. Although culture remains the gold standard technique and most widely used diagnostic approach in clinical microbiology, it is inherently limited by the fastidious growth requirements of certain fungal species, prior antifungal or antibiotic exposure that may suppress viable growth, and the overgrowth of faster-growing organisms that can mask slower-growing or less competitive species (Vanzolini and Magnani, 2024). In contrast, molecular techniques such as internal transcribed spacer (ITS) sequencing can detect non-viable or non-culturable organisms directly from clinical specimens and have been shown in other infection contexts to reveal substantially greater fungal diversity than culture alone (Lion, 2017; Vanzolini and Magnani, 2024). In this review, only 5 of 26 included studies employed molecular identification methods, while culture-based approaches remained the most frequently used diagnostic method (**Table 5**). Consequently, the species distributions reported in this review, particularly the relative rarity of certain yeasts and molds, may reflect diagnostic limitations rather than true low prevalence, and the actual burden and diversity of fungal pathogens in DFUs is likely underrepresented by the current evidence base. This underscores the need for future studies to incorporate molecular diagnostic techniques alongside conventional culture to more accurately characterize the fungal microbiome of DFUs.

Genus-level identification alone carries direct clinical implications, as species within the same genus can differ substantially in virulence, clinical significance, and antifungal susceptibility. Notably, certain *Aspergillus* spp., including *A. nidulans*, *A. terreus*, and *A. flavus*, exhibit intrinsic resistance to amphotericin B, in contrast to *A. fumigatus*, which is generally amphotericin B-susceptible (Lass-Flörl et al., 2021). Some of the included studies reported isolates only at the genus level, without species-level differentiation, among filamentous fungi (*Aspergillus* spp. and *Fusarium* spp., **Table 3**) and among dermatophytes (*Trichophyton* spp. and *Microsporum* spp., **Table 4**). Consequently, treatment decisions based solely on genus-level identification may be suboptimal or ineffective, emphasizing the importance of species-level identification by molecular techniques as a clinically actionable step toward optimizing antifungal treatment selection in DFUs.

Beyond individual fungal prevalence, the polymicrobial nature of most included studies (69.2%) necessarily needs to be taken into consideration in the fungal-bacterial interactions within DFU biofilms. For instance, *C. albicans* has been shown to secrete quorum-sensing molecules that promote bacterial biofilm formation, whereas certain bacterial metabolites can enhance fungal virulence, increasing resistance to both antifungal and antibiotic agents. Additionally, these mixed biofilms might serve as a reservoir for the horizontal transfer of resistance genes that confer resistance to antibiotics between species (Dhamgaye et al., 2016; Short et al., 2023). Moreover, experimental data show that mixed bacterial-fungal communities exhibit significantly higher antimicrobial tolerance than single-species biofilms (Dhamgaye et al., 2016). Here, species-specific interactions in DFU isolates can be bidirectional, with certain bacterial metabolites increasing rather than decreasing fungal susceptibility to antifungal agents. None of the included studies investigated these mechanistic interactions, which are a crucial area for future research beyond straightforward co-isolation reporting (Short et al., 2023; Allkja et al., 2025).

Case-based research accounted for 11.5% of our studies, which offered valuable clinical insights into the emerging role of fungal pathogens in DFUs, particularly atypical infection with rare fungi. The case reports specifically focused on severe invasive infections by uncommon fungi, such as *Trichosporon asahii*, *Cutaneotrichosporon debeurmannianum*, and *Fusarium oxysporum* (**Table 1**). Despite the case reports’ limited generalizability, this highlights the necessity of raising clinical awareness of the full spectrum of fungal involvement in DFIs. A critical limitation across the included studies is the frequent lack of distinction between fungal colonization and true infection. Most included investigations reported fungal isolation from DFUs based solely on culture or microscopic examination, without any corroborating clinical criteria or histopathological evidence of tissue invasion. Only three of the 26 included studies (11.5%), all case reports, used histological staining techniques (e.g., hematoxylin and eosin or Grocott methenamine silver stain) that provided direct evidence of invasive fungal disease (**Table 5**). This ambiguity in fungal accurate interpretation of the true clinical burden of fungal pathogens in DFUs emphasizes the necessity for standardized diagnostic criteria in future studies that explicitly distinguish colonization from active infection. This uncertainty is further exacerbated in polymicrobial wounds, where the majority of included studies reported co-isolation of bacterial and fungal organisms (n = 18, 69.2%). It is particularly challenging to determine the pathogenic contribution of any single isolated fungal species in such settings because its presence may reflect colonization within a complex microbial community rather than an independent driver of infection or delayed healing.

Antifungal drug resistance is a recognized global concern (Denning, 2024; Gurajala and Challapilla, 2026). However, our analysis shows that antifungal susceptibility testing (AFST) was performed in only 3 of 26 included studies (11.5%), which substantially limits the ability to draw firm conclusions regarding resistance patterns in this population. Within this limited dataset, resistance rates among *Candida* spp. ranged from 7.7% to 23.0% for select antifungal agents (**Table 6**); however, these findings should be interpreted as preliminary observations from a small number of studies rather than established resistance trends, given the cross-sectional nature of the available data and the absence of standardized susceptibility testing protocols across studies. This scarcity of AFST data represents a significant gap in the current literature, which limits the optimization of future antifungal therapy, particularly for immunocompromised diabetic patients with chronic wounds and repeated antimicrobial exposure, and underscores the absence of licensed antifungal vaccines for humans (Rodrigues and Nosanchuk, 2023). Standardized, prospective antifungal susceptibility testing is therefore needed to establish reliable resistance trends and inform future therapeutic strategies.

The included studies demonstrated considerable methodological heterogeneity across multiple domains, which limits the comparability of findings and should be considered when interpreting the overall findings. Study designs ranged from prospective (53.8%), retrospective studies (34.6%), and case reports (11.5%). A thought that the clinical information of case reports cannot provide reliable prevalence estimates, and were analyzed separately where possible. Patient populations also differed markedly in sample size, diabetes duration, glycemic control, and geographic origin, wound severity, and prior antimicrobial or antifungal exposure, all which factors that were inconsistently reported across studies may independently influence fungal colonization or infection risk. Specimen collection methods (deep tissue biopsy, wound swabs, or curettage) varied across the included studies, which influenced fungal detection rates, as these sampling techniques differ in their sensitivity for fungal recovery. Similarly, laboratory identification techniques were inconsistent, with conventional culture-based methods being most frequently used (n = 14), often alongside advanced techniques such as MALDI-TOF mass spectrometry and VITEK automated systems (n = 13), while molecular methods, such as ITS sequencing, were applied in only 5 studies, and histopathological confirmation was limited to two case-based studies (**Table 5**). Since diagnostic techniques differ substantially in sensitivity and specificity — with molecular methods generally detecting greater fungal diversity than culture-based approaches alone — pooling prevalence estimates across studies using different methodologies may introduce interpretation bias and should be viewed as descriptive rather than directly comparable. Collectively, this heterogeneity means that the pooled prevalence and species distribution figures reported in this review should be interpreted as a broad descriptive synthesis rather than precise epidemiological estimates, and any comparisons between studies, regions, or fungal species should be made with appropriate caution. Specimen collection technique also differed across studies, which may have influenced fungal recovery rates, as deep tissue biopsy is often more sensitive than superficial swabs or curettage in detecting fungal species. However, the majority of the included studies did not identify the technique utilized, restricting direct comparison.

Regional differences in fungal species distribution were also apparent across the included studies, although these should be interpreted cautiously due to the small number of investigations per region. Several studies from South Asia (India, Pakistan, Iraq) reported a predominance of non-*albicans Candida* species, particularly *C. tropicalis*, over *C. albicans*; for example, *C. albicans* accounted for only 13.3% of *Candida* isolates in an Iraqi cohort, compared to 58.4% for non-*albicans Candida* species in the same study (Al-Chalabi et al., 2024). In contrast, a Kenyan cohort reported *C. albicans* as the predominant species (75.0%) (Musyoki et al., 2022), while Chinese cohorts showed more varied patterns, with *C. parapsilosis* dominating (44.4%) and *C. albicans*, *C. tropicalis*, and *C. parapsilosis* distributed more evenly (Shi et al., 2022). Geographical variability, including climatic and environmental differences, variation in local antifungal prescribing practices, and variations in healthcare-associated exposure, has been suggested as a potential contributor to such regional variation in *Candida* epidemiology more generally. The included studies did not directly investigate the underlying drivers of these patterns, and the small number of studies per region precludes definitive conclusions. However, these findings imply that when evaluating fungal prevalence data in DFUs, regional epidemiological context may be important, and aggregated species distributions (**Table 2**) should not be assumed to generalize uniformly across geographic settings.

Beyond diagnostic considerations, failure to recognize potential fungal involvement in DFUs may contribute to prolonged and potentially unnecessary antibacterial therapy. Conversely, empirical antifungal therapy in the absence of confirmatory diagnostic testing may expose patients to unnecessary treatment and increase selection pressure for antifungal resistance. Incorporating fungal-specific diagnostic algorithms into the management of chronic non-healing DFUs may support more targeted antimicrobial therapy and strengthen antimicrobial stewardship in this patient population.

Notably, the included studies were concentrated in a limited number of countries (n = 14), with China contributing the highest number (**Figure 3**), while Saudi Arabia remains underrepresented. This is particularly important given the substantial national burden of diabetic foot complications, which parallels the rising prevalence of diabetes mellitus in the country (Al Dawish et al., 2016; Siddiqui et al., 2021; Zaki et al., 2024). Despite this, there is a clear lack of studies specifically addressing fungal involvement in DFUs, indicating a critical and underexplored research gap. In this review, only two studies from Saudi Arabia were identified, representing approximately 8.0% of the included literature. Both studies were primarily designed to investigate bacterial epidemiology, with fungal findings reported incidentally rather than through systematic investigation (Saeb et al., 2021; Alkhatieb et al., 2024).

For instance, although Saeb et al. employed advanced diagnostic approaches, the study’s focus remained on bacterial community profiling. The detection of *Candida* spp. appeared secondary and was not driven by a predefined objective to investigate fungal pathogens (Saeb et al., 2021). Moreover, the use of 16S rRNA sequencing—targeting bacterial DNA exclusively—further confirms the absence of a dedicated fungal analytical framework (**Table 5**). Similarly, Alkhatieb et al. adopted a retrospective design centered on bacterial characterization, with limited reporting of fungal isolates (e.g., *Candida* spp. at 2.1%) and no species-level identification (Alkhatieb et al., 2024). The absence of detailed fungal characterization in these studies likely reflects constraints in routine diagnostic workflows, where fungal detection is not systematically prioritized. This limitation is further accentuated in retrospective designs, where data availability is inherently restricted.

Collectively, these findings suggest that the current evidence base may underestimate the contribution of fungi to DFUs in Saudi Arabia, resulting in an incomplete understanding of the infectious landscape. Addressing this gap aligns with national research priorities aimed at improving outcomes in diabetes-related complications. Given that this observation is based on only two studies, both of which have a primary focus on bacterial rather than fungal epidemiology, any conclusions regarding the true DFU-fungal involvement in Saudi Arabia should be considered preliminary rather than definitive. Future research should prioritize fungal-focused DFU investigations, incorporating AFST, molecular identification methods such as ITS sequencing, and integration of fungal diagnostics into routine clinical workflows. In parallel, the development of national or regional fungal databases and their linkage with diabetic foot surveillance systems will be essential for tracking epidemiological trends, monitoring antifungal resistance, and informing evidence-based management strategies.

In this study, we acknowledge several limitations. There was considerable variability among the included studies in terms of study design, patient populations, microbiological diagnostic methodologies, and geographical regions, and most studies did not adjust for clinical confounders such as prior antimicrobial exposure, osteomyelitis, vascular insufficiency, ulcer severity, or immunosuppressive therapy. The literature search was limited to PubMed, Web of Science, and Google Scholar databases; other databases such as Scopus, Embase, and the Cochrane Library were not searched, which may have resulted in the omission of additional relevant studies. Restricting the search to studies published between 2021 and 2025 was intended to provide an updated synthesis of recent advances in fungal diagnosis and antifungal susceptibility testing; however, this approach may have excluded earlier relevant evidence on fungal involvement in DFUs and limited the overall comprehensiveness of available evidence. In addition, this review may also be influenced by publication bias, as studies reporting notable fungal prevalence are more likely to be published, potentially inflating the perceived clinical significance of fungal involvement in DFUs; language bias, as only English-language publications were included; and selective reporting bias, as outcomes were reported inconsistently across the included studies. These factors may have altered the overall representation and interpretation of the findings. Additionally, one included study (Saeb et al., 2021) remains a preprint without peer-reviewed publication, which may represent an additional source of bias. An uneven geographic representation with limited data from certain regions, including Saudi Arabia, may restrict the generalizability of the findings. Although case reports provide important clinical insights, their inclusion limits epidemiological generalizability. The absence of fungi in routine clinical microbiological assays and the lack of a universal diagnostic standardized system for fungi identification led to an underestimation of fungi diversity and prevalence in DFUs. Most included studies did not consistently distinguish between fungal colonization and true infection, which may have affected the interpretation of fungal involvement in DFUs. In addition, AFST was performed in only 3 of 26 included studies, and testing protocols were not standardized across these studies. This represents a major limitation, as it substantially restricts the reliability of conclusions about antifungal resistance patterns in this population. As a result, the resistance rates presented in this review should be regarded as preliminary rather than representative. Furthermore, none of the three studies reporting quantitative AFST explicitly specified the interpretive breakpoint standards used to classify isolates as susceptible or resistant (e.g., EUCAST guidelines or CLSI), despite using different testing. Because CLSI and EUCAST breakpoints can differ for certain species-drug combinations, the absence of explicit breakpoint reporting limits direct comparison of resistance rates across studies. Overall, these limitations emphasize the necessity of paying more attention to further investigating fungi in diabetic ulceration and standardized fungal diagnostic algorithms, and conducting large-scale comprehensive studies focusing specifically on fungal pathogens in chronic wounds, particularly in light of the challenges of resistance to antifungal agents.

**Figure 4:**
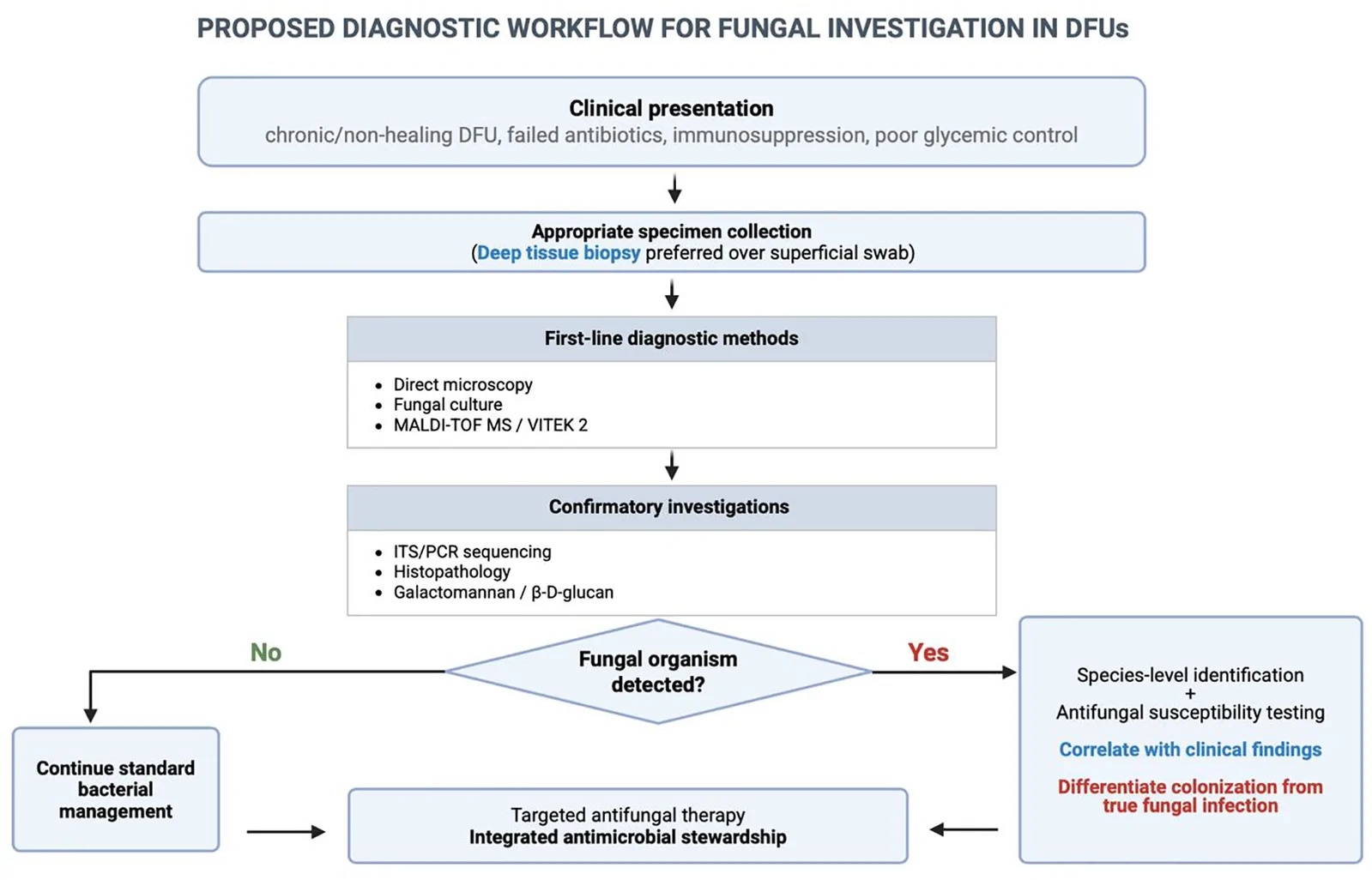
Proposed diagnostic workflow for fungal investigation in DFUs. The workflow integrates clinical assessment, specimen collection, and sequential laboratory testing to support evidence-informed evaluation of potential fungal involvement in DFUs. The workflow was created using BioRender (https://biorender.com).

To facilitate the clinical application of these findings, we propose a diagnostic workflow (**Figure 4**) that integrates clinical assessment, appropriate specimen collection, and stepwise diagnostic testing to support targeted fungal investigation and guide management in DFUs.

## 5 Conclusion

This study suggests a potentially important role for fungi in DFUs that warrants confirmation through well-designed, prospective, multicenter studies using standardized diagnostic protocols. Given the observational nature of the current evidence, growing antifungal resistance among fungal isolates raises concern and merits continued surveillance. The scarcity of studies in this field and the limited availability of updated information on DFU fungal infections pose considerable challenges to managing diabetic ulcers. Coordinated national and international efforts are urgently needed, including continuous monitoring, standardized diagnostic and large surveillance studies, to address this worldwide public health challenge.
