# Supplementary material for: Fungal detection, diagnostic approaches, and antifungal susceptibility in diabetic foot ulcers: a systematic review of recent evidence
Source: Front Fungal Biol. 2026 Sep 4;7:1915109. doi: 10.3389/ffunb.2026.1915109 (PMC13585981; doi:10.3389/ffunb.2026.1915109)
Supplement: Supplementary file 1 [file DataSheet1.pdf]

Investigating the Important and Overlooked Role of Fungi in Diabetic Foot Ulcers: A Systematic Review

Supplementary:

Table S1. Search strategies used across databases

Database	Search Strategy
**PubMed**	("diabetic foot ulcer"[MeSH Terms] OR "diabetic foot ulcer*" OR "diabetic foot infection*" OR "diabetic foot wound*") AND ("fung*" OR "mycosis" OR "mycoses" OR "Candida*" OR "Aspergillus*" OR "fungal coloniz*" OR "mold*" OR "mould*") AND ("2021/01/01"[Date - Publication] : "2025/12/31"[Date - Publication])
**Web of Science**	TS = (("diabetic foot ulcer*" OR "diabetic foot infection*" OR "diabetic foot wound*" OR "DFU" OR "DFI") AND ("fung*" OR "mycosis" OR "mycoses" OR "Candida*" OR "Aspergillus*" OR "fungal coloniz*" OR "mold*" OR "mould*" OR "tinea" OR "dermatophyt*"))
**Google Scholar**	("diabetic foot ulcer" OR "diabetic foot infection" OR "diabetic foot wound") AND (fungi OR fungal OR mycosis OR Candida OR Aspergillus OR mold) after:2020

Table S2. Quality assessment of included studies using the JBI Appraisal Tools

Study Number	Author, Year	Q1	Q2	Q3	Q4	Q5	Q6	Q7	Q8	Total Score
1.	João et al., 2021 [39]	Y	U	Y	Y	Y	Y	Y	Y	7
2.	Saeb et al., 2021 [31]	Y	Y	Y	Y	Y	N	Y	Y	7
3.	Chai et al., 2021 [40]	Y	Y	Y	Y	Y	Y	N	Y	7
4.	Li et al., 2022 [41]	Y	Y	Y	Y	Y	Y	Y	Y	8
5.	Yoo et al., 2022 [42]	Y	Y	Y	Y	Y	N	N	Y	7
6.	Musyoki et al., 2022 [43]	Y	Y	Y	Y	Y	Y	Y	Y	8
7.	Kandregula et al., 2022 [44]	Y	Y	Y	Y	Y	Y	Y	Y	8
8.	Moslemi et al., 2023 [45]	U	Y	Y	Y	Y	Y	Y	Y	7
9.	J. Liu et al., 2023 [46]	Y	Y	Y	Y	N	Y	Y	Y	7
10.	Devasia et al., 2023 [47]	U	Y	Y	Y	Y	Y	U	Y	6
11.	Shalaby et al., 2023 [48]	U	Y	Y	Y	Y	Y	Y	Y	7
12.	Helal, S.K., et al., 2023 [49]	U	Y	Y	Y	Y	Y	U	Y	6
13.	Kibunto, P., et al., 2023 [50]	U	Y	Y	Y	Y	Y	Y	Y	7
14.	Shi, M.-L., et al, 2023 [51]	Y	Y	Y	Y	Y	Y	Y	Y	8
15.	W. Liu et al., 2023 [46]	Y	Y	Y	Y	Y	Y	Y	Y	8
16.	Azharuddin et al., 2023[52]	Y	Y	Y	Y	Y	Y	Y	Y	8
17.	Coşkun et al., 2024 [53]	U	Y	Y	Y	Y	Y	Y	Y	7
18.	Frost et al., 2024 [54]	Y	U	Y	Y	Y	Y	N	Y	6
19.	Alkhatieb et al., 2024 [36]	Y	Y	Y	Y	Y	Y	Y	Y	8
20.	Al-Chalabi, F.A., et al., 2024 [55]	U	Y	Y	Y	Y	Y	Y	Y	7
21.	Mustafa, A.M., et al., 2024 [56]	U	Y	Y	Y	N	Y	Y	Y	6
22.	Xie et al., 2024 [57]	U	Y	Y	Y	Y	Y	N	Y	6
23.	Gonçalves et al., 2024 [58]	Y	Y	Y	Y	Y	Y	Y	Y	8
24.	Allkja et al., 2025 [59]	U	Y	Y	Y	Y	Y	Y	Y	7
25.	Zhang et al., 2025 [60]	Y	Y	Y	Y	Y	Y	Y	Y	8
26.	Kainat et al., 2025 [61]	Y	Y	Y	Y	Y	Y	Y	Y	8
